# (2-Amino-5-chloro­benzene­sulfonato-κ*O*)bis­(3-methyl­isoquinoline-κ*N*)silver(I)

**DOI:** 10.1107/S1600536810009220

**Published:** 2010-03-17

**Authors:** Qiang Liu, Li Feng, Yu-Jie Li, Xian-Wu Dong

**Affiliations:** a Jilin Agricultural Science and Technology College, Jilin 132101, People’s Republic of China

## Abstract

The title compound, [Ag(C_6_H_5_ClNO_3_S)(C_10_H_9_N)_2_], crystallizes with two independent mol­ecules in the asymmetric unit. The Ag^+^ cation is three-coordinated by one O atom from the 2-amino-5-chloro­benzene­sulfonate anion and two N atoms from two different 3-methyl­isoquinoline ligands in a slightly distorted trigonal-planar geometry. In the crystal, network of inter­molecular N—H⋯O hydrogen-bonding inter­actions generates a chain along [100].

## Related literature

For related structures, see: Li *et al.* (2007 ▶); Mišek *et al.* (2008 ▶); Wang *et al.* (2007 ▶); Shimizu *et al.* (1999 ▶).
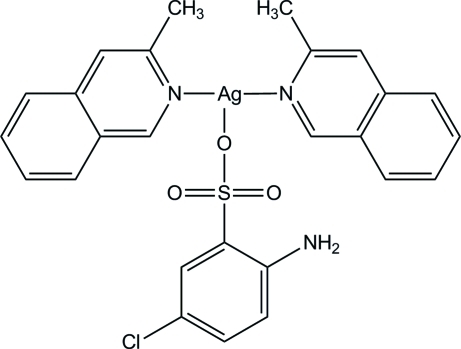


## Experimental

### 

#### Crystal data

[Ag(C_6_H_5_ClNO_3_S)(C_10_H_9_N)_2_]
*M* *_r_* = 600.68
Orthorhombic, 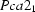

*a* = 21.184 (2) Å
*b* = 14.7095 (10) Å
*c* = 15.6602 (10) Å
*V* = 4879.8 (6) Å^3^
*Z* = 8
Mo *K*α radiation
μ = 1.06 mm^−1^
*T* = 293 K
0.23 × 0.19 × 0.17 mm

#### Data collection

Bruker SMART APEX CCD diffractometer
Absorption correction: multiscan (*SADABS*; Sheldrick, 1996 ▶) *T*_min_ = 0.779, *T*_max_ = 0.831
13070 measured reflections
8349 independent reflections
3592 reflections with *I* > 2σ(*I*)
*R*_int_ = 0.086

#### Refinement

*R*[*F* ^2^ > 2σ(*F* ^2^)] = 0.056
*wR*(*F* ^2^) = 0.124
*S* = 0.81
8349 reflections
641 parameters
16 restraints
H atoms treated by a mixture of independent and constrained refinement
Δρ_max_ = 0.85 e Å^−3^
Δρ_min_ = −0.67 e Å^−3^
Absolute structure: Flack (1983 ▶), 3745 Friedel pairs
Flack parameter: −0.06 (4)

### 

Data collection: *SMART* (Bruker, 2001 ▶); cell refinement: *SAINT* (Bruker, 2001 ▶); data reduction: *SAINT*; program(s) used to solve structure: *SHELXS97* (Sheldrick, 2008 ▶); program(s) used to refine structure: *SHELXL97* (Sheldrick, 2008 ▶); molecular graphics: *SHELXTL* (Sheldrick, 2008 ▶); software used to prepare material for publication: *SHELXL97*.

## Supplementary Material

Crystal structure: contains datablocks I, global. DOI: 10.1107/S1600536810009220/zq2031sup1.cif

Structure factors: contains datablocks I. DOI: 10.1107/S1600536810009220/zq2031Isup2.hkl

Additional supplementary materials:  crystallographic information; 3D view; checkCIF report

## Figures and Tables

**Table 1:** Hydrogen-bond geometry (Å, °)

*D*—H⋯*A*	*D*—H	H⋯*A*	*D*⋯*A*	*D*—H⋯*A*
N6—H6*NA*⋯O8	0.83	2.35	2.844 (12)	118
N5—H5*NA*⋯O9	0.84 (3)	2.20 (5)	3.006 (13)	159 (11)
N5—H5*NB*⋯O5	0.83 (3)	2.48 (6)	2.973 (14)	119 (4)
N6—H6*NB*⋯O4^i^	0.84	2.30	3.126 (13)	168

Symmetry code: (i) 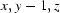.

**Fig. 1.**
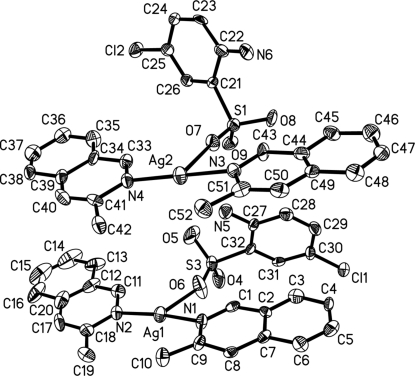
ORTEP diagram of the title compound, showing 30% probability displacement ellipsoids. H atoms have been omitted for clarity.

**Fig. 2.**
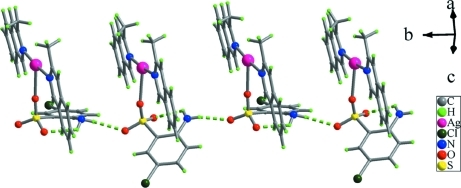
Representation of the one-dimensional chain formed by intermolecular N—H···O hydrogen bonding interactions.

## supplementary crystallographic information

### Comment

In the crystal structure of the title compound, each Ag^+^ cation is three-coordinated by one O atom from the 2-amino-5-chlorobenzenesulfonate anion and two N atoms from two different 3-methylisoquinoline ligands in a slightly distorted trigonal-planar geometry (Fig. 1). The Ag—O distances of 2.645 (7) Å and 2.630 (9) Å, and the Ag—N distances in the range 2.158 (10) - 2.200 (9) Å are similar to whose within the previous reports (Li *et al.*, 2007; Mišek *et al.*, 2008; Shimizu *et al.*, 1999; Wang *et al.*, 2007). A network of intermolecular N—H···O hydrogen bonding interactions generates an one-dimensional chain (Fig 2).

### Experimental

An aqueous solution (10 ml) of 2-amino-5-chlorobenzene-1-sulfonic acid (0.041 g, 0.5 mmol) was addded to solid Ag_2_CO_3_ (0.069 g, 0.25 mmol) and stirred for several minutes until no further CO_2_ was given off; 3-methylisoquinoline (0.0715 g, 0.5 mmol) in methanol (5 ml) was then added and a white precipitate formed. The precipitate was dissolved by dropwise addition of an aqueous solution of NH_3_ (14 M). Crystals of the title compound were obtained by evaporation of the solution for several days at room temperature.

### Refinement

The C-bound H atoms were geometrically positioned (C—H 0.93 Å) and refined using a riding model with U_iso_ = 1.2U_eq_ (C). The H atoms of the amino groups were located in Fourier difference maps: the two H atoms bound to N5 were refined with distance restraints (N—H = 0.85 Å) while the other two H atoms (N6) were fully refined (all four H atoms with U_iso_ = 1.2U_eq_ (N)).

### Crystal data

**Table:**

[Ag(C_6_H_5_ClNO_3_S)(C_10_H_9_N)_2_]	*D*_x_ = 1.635 Mg m^−^^3^
*M**_r_* = 600.68	Melting point: not measured K
Orthorhombic, *P**c**a*2_1_	Mo *K*α radiation, λ = 0.71073 Å
Hall symbol: P 2c -2ac	Cell parameters from 8349 reflections
*a* = 21.184 (2) Å	θ = 1.5–25.3°
*b* = 14.7095 (10) Å	µ = 1.06 mm^−^^1^
*c* = 15.6602 (10) Å	*T* = 293 K
*V* = 4879.8 (6) Å^3^	Block, colorless
*Z* = 8	0.23 × 0.19 × 0.17 mm
*F*(000) = 2432	

### Data collection

**Table:**

Bruker SMART APEX CCD diffractometer	8349 independent reflections
Radiation source: fine-focus sealed tube	3592 reflections with *I* > 2σ(*I*)
graphite	*R*_int_ = 0.086
phi and ω scans	θ_max_ = 25.3°, θ_min_ = 1.7°
Absorption correction: multi-scan (*SADABS*; Sheldrick, 1996)	*h* = −25→6
*T*_min_ = 0.779, *T*_max_ = 0.831	*k* = −16→17
13070 measured reflections	*l* = −18→18

### Refinement

**Table:**

Refinement on *F*^2^	Secondary atom site location: difference Fourier map
Least-squares matrix: full	Hydrogen site location: inferred from neighbouring sites
*R*[*F*^2^ > 2σ(*F*^2^)] = 0.056	H atoms treated by a mixture of independent and constrained refinement
*wR*(*F*^2^) = 0.124	*w* = 1/[σ^2^(*F*_o_^2^) + (0.029*P*)^2^] where *P* = (*F*_o_^2^ + 2*F*_c_^2^)/3
*S* = 0.81	(Δ/σ)_max_ = 0.001
8349 reflections	Δρ_max_ = 0.85 e Å^−^^3^
641 parameters	Δρ_min_ = −0.67 e Å^−^^3^
16 restraints	Absolute structure: Flack (1983), 3745 Friedel pairs
Primary atom site location: structure-invariant direct methods	Flack parameter: −0.06 (4)

### Special details

**Table:**

Geometry. All esds (except the esd in the dihedral angle between two l.s. planes) are estimated using the full covariance matrix. The cell esds are taken into account individually in the estimation of esds in distances, angles and torsion angles; correlations between esds in cell parameters are only used when they are defined by crystal symmetry. An approximate (isotropic) treatment of cell esds is used for estimating esds involving l.s. planes.
Refinement. Refinement of *F*^2^ against ALL reflections. The weighted *R*-factor wR and goodness of fit *S* are based on *F*^2^, conventional *R*-factors *R* are based on *F*, with *F* set to zero for negative *F*^2^. The threshold expression of *F*^2^ > σ(*F*^2^) is used only for calculating *R*-factors(gt) etc. and is not relevant to the choice of reflections for refinement. *R*-factors based on *F*^2^ are statistically about twice as large as those based on *F*, and *R*- factors based on ALL data will be even larger.

### Fractional atomic coordinates and isotropic or equivalent isotropic displacement parameters (Å^2^)

**Table:**

	*x*	*y*	*z*	*U*_iso_*/*U*_eq_	
Ag1	0.25032 (4)	0.63381 (6)	0.06884 (7)	0.0513 (2)	
Ag2	0.27564 (4)	0.13421 (6)	0.07551 (6)	0.0542 (3)	
C1	0.2390 (5)	0.5454 (7)	0.2400 (7)	0.038 (3)	
H1	0.1969	0.5554	0.2260	0.046*	
C2	0.2542 (7)	0.5093 (6)	0.3221 (10)	0.035 (3)	
C3	0.2067 (6)	0.4903 (8)	0.3795 (8)	0.053 (4)	
H3	0.1644	0.4977	0.3653	0.063*	
C4	0.2251 (7)	0.4594 (8)	0.4600 (8)	0.055 (4)	
H4	0.1940	0.4451	0.4997	0.066*	
C5	0.2896 (7)	0.4489 (8)	0.4835 (8)	0.057 (4)	
H5	0.3004	0.4291	0.5380	0.068*	
C6	0.3338 (5)	0.4678 (9)	0.4269 (8)	0.051 (4)	
H6	0.3759	0.4595	0.4418	0.061*	
C7	0.3186 (6)	0.5015 (8)	0.3409 (8)	0.038 (3)	
C8	0.3643 (5)	0.5205 (7)	0.2793 (7)	0.036 (3)	
H8	0.4070	0.5137	0.2918	0.044*	
C9	0.3454 (6)	0.5496 (8)	0.1992 (7)	0.044 (3)	
C10	0.3918 (5)	0.5668 (8)	0.1310 (7)	0.057 (4)	
H10A	0.3783	0.6177	0.0972	0.085*	
H10B	0.3953	0.5140	0.0953	0.085*	
H10C	0.4322	0.5800	0.1560	0.085*	
C11	0.1698 (6)	0.7127 (8)	−0.0731 (9)	0.051 (4)	
H11	0.1386	0.6934	−0.0355	0.062*	
C12	0.1514 (8)	0.7482 (9)	−0.1540 (11)	0.064 (4)	
C13	0.0880 (7)	0.7547 (9)	−0.1746 (10)	0.078 (5)	
H13	0.0573	0.7382	−0.1350	0.094*	
C14	0.0710 (10)	0.7853 (13)	−0.2528 (14)	0.121 (8)	
H14	0.0288	0.7917	−0.2680	0.146*	
C15	0.1197 (12)	0.8071 (14)	−0.3100 (14)	0.125 (10)	
H15	0.1088	0.8282	−0.3640	0.150*	
C16	0.1783 (10)	0.7993 (11)	−0.2917 (10)	0.098 (7)	
H16	0.2085	0.8139	−0.3326	0.118*	
C17	0.2604 (9)	0.7612 (8)	−0.1847 (11)	0.067 (5)	
H17	0.2922	0.7762	−0.2232	0.080*	
C18	0.2776 (6)	0.7318 (8)	−0.1035 (9)	0.045 (3)	
C19	0.3416 (6)	0.7216 (9)	−0.0721 (10)	0.066 (4)	
H19A	0.3441	0.7441	−0.0147	0.099*	
H19B	0.3700	0.7554	−0.1079	0.099*	
H19C	0.3531	0.6584	−0.0730	0.099*	
C20	0.1977 (10)	0.7693 (10)	−0.2111 (11)	0.076 (6)	
C21	0.0488 (5)	0.0799 (7)	0.0542 (7)	0.035 (3)	
C22	0.0473 (5)	−0.0178 (8)	0.0625 (10)	0.047 (3)	
C23	0.0203 (6)	−0.0640 (8)	−0.0051 (8)	0.049 (3)	
H95	0.0194	−0.1272	−0.0027	0.058*	
C24	−0.0052 (5)	−0.0228 (9)	−0.0756 (8)	0.045 (3)	
H94	−0.0241	−0.0574	−0.1183	0.054*	
C25	−0.0027 (5)	0.0701 (9)	−0.0824 (7)	0.042 (3)	
C26	0.0234 (5)	0.1205 (8)	−0.0160 (7)	0.037 (3)	
H92	0.0235	0.1836	−0.0197	0.045*	
C27	0.0584 (5)	0.4823 (8)	0.2184 (7)	0.036 (3)	
C28	0.0441 (5)	0.4330 (8)	0.2919 (8)	0.048 (3)	
H99	0.0462	0.3699	0.2897	0.058*	
C29	0.0268 (5)	0.4738 (8)	0.3682 (8)	0.049 (3)	
H100	0.0189	0.4390	0.4166	0.059*	
C30	0.0215 (5)	0.5683 (9)	0.3705 (7)	0.044 (3)	
C31	0.0314 (4)	0.6194 (7)	0.2970 (6)	0.036 (3)	
H102	0.0250	0.6819	0.2982	0.043*	
C32	0.0508 (5)	0.5780 (8)	0.2217 (7)	0.038 (3)	
C33	0.2104 (6)	0.2154 (8)	−0.0753 (7)	0.042 (3)	
H59	0.1767	0.2015	−0.0395	0.051*	
C34	0.1972 (7)	0.2484 (10)	−0.1602 (8)	0.045 (4)	
C35	0.1361 (6)	0.2610 (9)	−0.1908 (9)	0.059 (4)	
H57	0.1019	0.2500	−0.1552	0.071*	
C36	0.1260 (6)	0.2896 (9)	−0.2737 (9)	0.061 (4)	
H56	0.0854	0.2932	−0.2961	0.073*	
C37	0.1779 (7)	0.3126 (9)	−0.3222 (9)	0.064 (4)	
H55	0.1706	0.3358	−0.3765	0.077*	
C38	0.2379 (7)	0.3039 (8)	−0.2968 (8)	0.058 (4)	
H54	0.2708	0.3183	−0.3337	0.070*	
C39	0.2513 (7)	0.2718 (7)	−0.2111 (8)	0.049 (4)	
C40	0.3113 (7)	0.2583 (9)	−0.1766 (10)	0.057 (5)	
H52	0.3465	0.2710	−0.2100	0.069*	
C41	0.3200 (6)	0.2276 (8)	−0.0966 (9)	0.045 (3)	
C42	0.3828 (5)	0.2179 (9)	−0.0562 (8)	0.062 (4)	
H51A	0.3868	0.1581	−0.0324	0.093*	
H51B	0.3872	0.2623	−0.0117	0.093*	
H51C	0.4151	0.2272	−0.0984	0.093*	
C43	0.2361 (5)	0.0437 (8)	0.2403 (7)	0.048 (3)	
H69	0.1969	0.0537	0.2152	0.058*	
C44	0.2402 (7)	0.0130 (8)	0.3236 (10)	0.044 (3)	
C45	0.1819 (6)	−0.0041 (8)	0.3705 (9)	0.049 (4)	
H67	0.1431	0.0067	0.3447	0.059*	
C46	0.1844 (6)	−0.0367 (9)	0.4541 (9)	0.062 (4)	
H66	0.1478	−0.0471	0.4855	0.074*	
C47	0.2445 (7)	−0.0533 (8)	0.4889 (9)	0.059 (4)	
H65	0.2457	−0.0744	0.5448	0.070*	
C48	0.3016 (6)	−0.0415 (8)	0.4490 (8)	0.053 (4)	
H64	0.3399	−0.0537	0.4758	0.064*	
C49	0.2977 (7)	−0.0083 (8)	0.3613 (8)	0.043 (3)	
C50	0.3515 (6)	0.0072 (8)	0.3101 (9)	0.051 (4)	
H62	0.3910	−0.0050	0.3334	0.061*	
C51	0.3488 (6)	0.0382 (8)	0.2304 (9)	0.047 (3)	
C52	0.4045 (5)	0.0548 (9)	0.1719 (7)	0.059 (4)	
H61A	0.3990	0.1117	0.1427	0.089*	
H61B	0.4073	0.0065	0.1308	0.089*	
H61C	0.4426	0.0567	0.2050	0.089*	
Cl1	−0.00209 (15)	0.6221 (2)	0.46319 (18)	0.0590 (9)	
Cl2	−0.03193 (15)	0.1251 (2)	−0.17157 (18)	0.0583 (9)	
N1	0.2844 (4)	0.5654 (7)	0.1817 (6)	0.045 (3)	
N2	0.2299 (5)	0.7059 (6)	−0.0490 (6)	0.042 (3)	
N3	0.2901 (5)	0.0590 (6)	0.1954 (6)	0.041 (3)	
N4	0.2680 (5)	0.2041 (6)	−0.0464 (6)	0.043 (3)	
N5	0.0812 (6)	0.4377 (7)	0.1469 (6)	0.057 (3)	
N6	0.0779 (5)	−0.0619 (6)	0.1284 (6)	0.057 (3)	
H6NA	0.0606	−0.0434	0.1730	0.068*	
H6NB	0.0662	−0.1163	0.1266	0.068*	
O4	0.0392 (5)	0.7346 (6)	0.1514 (5)	0.079 (3)	
O5	0.0383 (5)	0.6065 (6)	0.0599 (7)	0.110 (4)	
O6	0.1352 (4)	0.6556 (8)	0.1279 (6)	0.099 (4)	
O7	0.1532 (3)	0.1489 (6)	0.1049 (4)	0.057 (2)	
O8	0.0777 (4)	0.1081 (5)	0.2150 (4)	0.056 (2)	
O9	0.0590 (4)	0.2375 (5)	0.1233 (5)	0.053 (2)	
S1	0.08796 (14)	0.1491 (2)	0.13082 (18)	0.0396 (8)	
S3	0.06739 (16)	0.6494 (2)	0.1324 (2)	0.0478 (9)	
H5NA	0.085 (6)	0.381 (2)	0.144 (5)	0.072*	
H5NB	0.088 (5)	0.460 (3)	0.099 (2)	0.072*	

### Atomic displacement parameters (Å^2^)

**Table:**

	*U*^11^	*U*^22^	*U*^33^	*U*^12^	*U*^13^	*U*^23^
Ag1	0.0592 (5)	0.0565 (5)	0.0382 (5)	0.0081 (5)	−0.0039 (7)	0.0097 (7)
Ag2	0.0612 (6)	0.0570 (6)	0.0445 (6)	0.0045 (5)	0.0007 (8)	0.0118 (8)
C1	0.044 (9)	0.028 (7)	0.042 (8)	0.004 (6)	−0.009 (6)	−0.014 (6)
C2	0.035 (7)	0.027 (6)	0.042 (7)	−0.005 (8)	0.002 (6)	0.006 (8)
C3	0.048 (9)	0.049 (10)	0.061 (10)	0.005 (7)	0.002 (8)	−0.008 (8)
C4	0.092 (9)	0.046 (9)	0.027 (7)	−0.025 (8)	0.005 (7)	−0.003 (7)
C5	0.087 (9)	0.043 (9)	0.041 (8)	0.007 (8)	0.000 (8)	−0.008 (7)
C6	0.035 (8)	0.058 (10)	0.059 (9)	0.004 (6)	−0.007 (7)	0.013 (8)
C7	0.044 (9)	0.033 (8)	0.036 (8)	0.008 (6)	−0.007 (6)	−0.007 (6)
C8	0.023 (7)	0.044 (8)	0.042 (8)	−0.002 (6)	−0.008 (6)	−0.009 (7)
C9	0.045 (9)	0.058 (9)	0.028 (7)	0.010 (7)	−0.007 (6)	−0.003 (7)
C10	0.055 (9)	0.071 (10)	0.044 (8)	0.015 (7)	0.006 (7)	0.011 (7)
C11	0.041 (9)	0.048 (9)	0.065 (10)	0.009 (7)	0.009 (7)	−0.007 (8)
C12	0.087 (13)	0.042 (10)	0.063 (11)	0.021 (9)	−0.038 (10)	−0.010 (9)
C13	0.094 (12)	0.049 (9)	0.091 (13)	0.001 (8)	−0.059 (10)	0.007 (9)
C14	0.17 (2)	0.071 (14)	0.122 (19)	0.045 (15)	−0.084 (15)	−0.009 (14)
C15	0.23 (3)	0.059 (13)	0.090 (16)	0.029 (18)	−0.077 (18)	0.014 (13)
C16	0.20 (2)	0.054 (11)	0.040 (11)	0.029 (13)	−0.033 (12)	0.002 (9)
C17	0.109 (16)	0.037 (8)	0.054 (11)	−0.003 (10)	0.045 (11)	0.001 (10)
C18	0.057 (10)	0.020 (8)	0.059 (10)	0.004 (7)	0.009 (8)	−0.007 (7)
C19	0.056 (10)	0.054 (10)	0.087 (12)	−0.008 (8)	0.006 (8)	0.009 (9)
C20	0.141 (19)	0.036 (10)	0.052 (13)	0.018 (11)	0.001 (13)	0.001 (9)
C21	0.046 (7)	0.035 (7)	0.024 (7)	−0.007 (5)	−0.005 (5)	−0.001 (6)
C22	0.054 (7)	0.039 (7)	0.050 (8)	−0.007 (5)	0.014 (8)	0.005 (8)
C23	0.070 (10)	0.017 (7)	0.059 (9)	−0.018 (7)	0.021 (8)	−0.004 (7)
C24	0.049 (8)	0.051 (10)	0.034 (7)	−0.014 (7)	0.006 (6)	−0.009 (7)
C25	0.031 (7)	0.051 (9)	0.044 (8)	−0.006 (6)	0.004 (6)	−0.006 (7)
C26	0.043 (7)	0.030 (7)	0.038 (7)	0.001 (6)	0.001 (6)	0.006 (7)
C27	0.032 (7)	0.047 (8)	0.028 (7)	0.002 (6)	−0.004 (5)	−0.001 (7)
C28	0.051 (8)	0.041 (8)	0.053 (9)	−0.003 (6)	−0.007 (6)	0.012 (7)
C29	0.064 (9)	0.040 (9)	0.044 (9)	−0.003 (7)	0.006 (7)	0.009 (7)
C30	0.046 (8)	0.055 (10)	0.031 (7)	0.002 (7)	−0.001 (6)	0.003 (6)
C31	0.035 (6)	0.030 (7)	0.042 (7)	−0.005 (5)	0.000 (5)	−0.009 (6)
C32	0.043 (8)	0.048 (9)	0.024 (7)	−0.003 (6)	−0.003 (5)	−0.009 (6)
C33	0.046 (9)	0.042 (8)	0.039 (8)	0.009 (6)	0.001 (6)	−0.001 (7)
C34	0.065 (11)	0.047 (9)	0.023 (8)	0.001 (8)	0.008 (7)	−0.008 (7)
C35	0.060 (10)	0.061 (10)	0.055 (10)	0.011 (8)	−0.004 (8)	0.012 (8)
C36	0.071 (11)	0.052 (10)	0.060 (10)	0.010 (8)	−0.012 (8)	−0.004 (9)
C37	0.102 (11)	0.037 (9)	0.053 (10)	0.008 (9)	−0.001 (9)	0.009 (8)
C38	0.097 (10)	0.036 (8)	0.042 (9)	−0.010 (8)	0.020 (8)	0.000 (7)
C39	0.090 (10)	0.023 (7)	0.035 (9)	0.003 (8)	0.019 (8)	−0.005 (6)
C40	0.054 (11)	0.043 (10)	0.075 (12)	−0.007 (8)	0.028 (9)	0.005 (9)
C41	0.043 (8)	0.028 (8)	0.063 (10)	−0.008 (6)	0.007 (7)	0.006 (7)
C42	0.064 (8)	0.076 (10)	0.047 (9)	−0.012 (8)	0.003 (7)	0.016 (8)
C43	0.047 (9)	0.063 (10)	0.034 (7)	0.005 (7)	−0.011 (6)	0.014 (7)
C44	0.053 (10)	0.038 (7)	0.042 (7)	−0.006 (7)	−0.006 (7)	0.003 (8)
C45	0.035 (9)	0.049 (10)	0.063 (10)	−0.008 (7)	−0.004 (7)	0.009 (8)
C46	0.060 (8)	0.058 (10)	0.067 (11)	−0.003 (8)	0.009 (7)	0.006 (9)
C47	0.091 (8)	0.035 (8)	0.050 (9)	−0.012 (8)	−0.007 (7)	0.011 (7)
C48	0.052 (7)	0.048 (9)	0.060 (10)	0.009 (7)	−0.021 (6)	0.008 (8)
C49	0.056 (9)	0.030 (8)	0.043 (9)	−0.006 (7)	−0.004 (7)	−0.002 (7)
C50	0.041 (9)	0.048 (9)	0.063 (10)	−0.003 (6)	−0.028 (8)	0.013 (8)
C51	0.037 (8)	0.042 (9)	0.062 (10)	0.014 (7)	−0.014 (7)	−0.011 (8)
C52	0.046 (8)	0.063 (10)	0.069 (10)	0.003 (7)	0.015 (7)	0.000 (8)
Cl1	0.069 (2)	0.071 (2)	0.0379 (17)	−0.0122 (19)	0.0097 (15)	−0.0056 (19)
Cl2	0.072 (2)	0.061 (2)	0.0418 (18)	0.0038 (19)	−0.0142 (15)	0.003 (2)
N1	0.039 (6)	0.064 (8)	0.030 (6)	0.010 (6)	0.003 (5)	0.001 (6)
N2	0.049 (7)	0.038 (6)	0.038 (6)	0.009 (5)	0.006 (5)	0.003 (5)
N3	0.050 (7)	0.035 (6)	0.039 (6)	−0.001 (5)	−0.004 (5)	0.008 (5)
N4	0.051 (7)	0.043 (7)	0.035 (6)	−0.001 (5)	0.005 (5)	0.007 (5)
N5	0.077 (8)	0.048 (8)	0.046 (7)	0.003 (7)	0.007 (6)	−0.004 (6)
N6	0.091 (9)	0.029 (7)	0.049 (7)	0.001 (6)	0.003 (6)	0.020 (5)
O4	0.122 (9)	0.057 (7)	0.057 (7)	0.016 (6)	0.022 (6)	0.023 (5)
O5	0.203 (11)	0.082 (7)	0.045 (6)	−0.020 (7)	−0.047 (8)	0.022 (6)
O6	0.061 (7)	0.154 (11)	0.083 (7)	0.017 (7)	0.022 (5)	0.055 (7)
O7	0.037 (4)	0.086 (6)	0.046 (5)	−0.010 (4)	0.002 (3)	0.006 (5)
O8	0.077 (6)	0.067 (6)	0.024 (5)	0.002 (5)	0.008 (4)	−0.006 (4)
O9	0.068 (6)	0.038 (5)	0.052 (6)	0.008 (4)	−0.011 (4)	−0.007 (5)
S1	0.0413 (19)	0.040 (2)	0.0370 (18)	0.0015 (16)	−0.0016 (14)	0.0003 (17)
S3	0.061 (2)	0.046 (2)	0.037 (2)	0.0097 (18)	0.0075 (17)	0.0083 (18)

### Geometric parameters (Å, °)

**Table:**

Ag1—N1	2.158 (10)	C27—C32	1.418 (14)
Ag1—N2	2.172 (9)	C28—C29	1.385 (15)
Ag1—O6	2.628 (9)	C28—H99	0.9300
Ag2—N4	2.175 (9)	C29—C30	1.395 (15)
Ag2—N3	2.200 (9)	C29—H100	0.9300
Ag2—O7	2.644 (7)	C30—C31	1.391 (14)
C1—N1	1.357 (13)	C30—Cl1	1.727 (12)
C1—C2	1.428 (17)	C31—C32	1.389 (13)
C1—H1	0.9300	C31—H102	0.9300
C2—C3	1.379 (18)	C32—S3	1.784 (12)
C2—C7	1.399 (17)	C33—N4	1.312 (13)
C3—C4	1.395 (16)	C33—C34	1.443 (16)
C3—H3	0.9300	C33—H59	0.9300
C4—C5	1.423 (16)	C34—C35	1.392 (16)
C4—H4	0.9300	C34—C39	1.438 (18)
C5—C6	1.320 (15)	C35—C36	1.381 (17)
C5—H5	0.9300	C35—H57	0.9300
C6—C7	1.471 (16)	C36—C37	1.377 (17)
C6—H6	0.9300	C36—H56	0.9300
C7—C8	1.396 (15)	C37—C38	1.339 (16)
C8—C9	1.385 (14)	C37—H55	0.9300
C8—H8	0.9300	C38—C39	1.449 (16)
C9—N1	1.341 (13)	C38—H54	0.9300
C9—C10	1.474 (14)	C39—C40	1.397 (18)
C10—H10A	0.9600	C40—C41	1.344 (17)
C10—H10B	0.9600	C40—H52	0.9300
C10—H10C	0.9600	C41—N4	1.396 (14)
C11—N2	1.331 (13)	C41—C42	1.480 (15)
C11—C12	1.426 (18)	C42—H51A	0.9600
C11—H11	0.9300	C42—H51B	0.9600
C12—C20	1.36 (2)	C42—H51C	0.9600
C12—C13	1.385 (17)	C43—N3	1.361 (13)
C13—C14	1.35 (2)	C43—C44	1.383 (17)
C13—H13	0.9300	C43—H69	0.9300
C14—C15	1.40 (3)	C44—C49	1.390 (19)
C14—H14	0.9300	C44—C45	1.459 (17)
C15—C16	1.28 (2)	C45—C46	1.395 (16)
C15—H15	0.9300	C45—H67	0.9300
C16—C20	1.40 (2)	C46—C47	1.407 (17)
C16—H16	0.9300	C46—H66	0.9300
C17—C18	1.392 (19)	C47—C48	1.372 (16)
C17—C20	1.40 (2)	C47—H65	0.9300
C17—H17	0.9300	C48—C49	1.460 (17)
C18—N2	1.377 (14)	C48—H64	0.9300
C18—C19	1.450 (16)	C49—C50	1.412 (16)
C19—H19A	0.9600	C50—C51	1.330 (16)
C19—H19B	0.9600	C50—H62	0.9300
C19—H19C	0.9600	C51—N3	1.394 (13)
C21—C26	1.363 (14)	C51—C52	1.514 (15)
C21—C22	1.443 (14)	C52—H61A	0.9600
C21—S1	1.779 (10)	C52—H61B	0.9600
C22—N6	1.381 (15)	C52—H61C	0.9600
C22—C23	1.383 (16)	N5—H5NA	0.84 (3)
C23—C24	1.371 (15)	N5—H5NB	0.83 (3)
C23—H95	0.9300	N6—H6NA	0.8343
C24—C25	1.372 (15)	N6—H6NB	0.8395
C24—H94	0.9300	O4—S3	1.419 (9)
C25—C26	1.391 (14)	O5—S3	1.437 (10)
C25—Cl2	1.729 (12)	O6—S3	1.441 (9)
C26—H92	0.9300	O7—S1	1.440 (7)
C27—N5	1.384 (13)	O8—S1	1.466 (8)
C27—C28	1.394 (14)	O9—S1	1.442 (8)
			
N1—Ag1—N2	172.0 (4)	C32—C31—H102	119.6
N1—Ag1—O6	94.5 (3)	C30—C31—H102	119.6
N2—Ag1—O6	93.1 (3)	C31—C32—C27	120.0 (11)
N4—Ag2—N3	175.7 (4)	C31—C32—S3	117.8 (9)
N4—Ag2—O7	92.4 (3)	C27—C32—S3	122.2 (9)
N3—Ag2—O7	91.6 (3)	N4—C33—C34	122.7 (12)
N1—C1—C2	121.8 (11)	N4—C33—H59	118.7
N1—C1—H1	119.1	C34—C33—H59	118.7
C2—C1—H1	119.1	C35—C34—C39	121.1 (14)
C3—C2—C7	123.9 (13)	C35—C34—C33	122.9 (13)
C3—C2—C1	119.9 (13)	C39—C34—C33	116.0 (13)
C7—C2—C1	116.1 (12)	C36—C35—C34	120.6 (14)
C2—C3—C4	116.8 (13)	C36—C35—H57	119.7
C2—C3—H3	121.6	C34—C35—H57	119.7
C4—C3—H3	121.6	C37—C36—C35	118.0 (13)
C3—C4—C5	122.5 (12)	C37—C36—H56	121.0
C3—C4—H4	118.8	C35—C36—H56	121.0
C5—C4—H4	118.8	C38—C37—C36	124.7 (14)
C6—C5—C4	119.0 (13)	C38—C37—H55	117.6
C6—C5—H5	120.5	C36—C37—H55	117.6
C4—C5—H5	120.5	C37—C38—C39	119.5 (13)
C5—C6—C7	122.0 (12)	C37—C38—H54	120.3
C5—C6—H6	119.0	C39—C38—H54	120.3
C7—C6—H6	119.0	C40—C39—C34	118.5 (13)
C8—C7—C2	121.0 (12)	C40—C39—C38	125.6 (13)
C8—C7—C6	123.2 (11)	C34—C39—C38	115.8 (14)
C2—C7—C6	115.7 (12)	C41—C40—C39	122.2 (13)
C9—C8—C7	119.1 (11)	C41—C40—H52	118.9
C9—C8—H8	120.4	C39—C40—H52	118.9
C7—C8—H8	120.4	C40—C41—N4	120.0 (12)
N1—C9—C8	121.2 (11)	C40—C41—C42	123.6 (13)
N1—C9—C10	117.7 (10)	N4—C41—C42	116.4 (12)
C8—C9—C10	121.1 (11)	C41—C42—H51A	109.5
C9—C10—H10A	109.5	C41—C42—H51B	109.5
C9—C10—H10B	109.5	H51A—C42—H51B	109.5
H10A—C10—H10B	109.5	C41—C42—H51C	109.5
C9—C10—H10C	109.5	H51A—C42—H51C	109.5
H10A—C10—H10C	109.5	H51B—C42—H51C	109.5
H10B—C10—H10C	109.5	N3—C43—C44	119.3 (11)
N2—C11—C12	122.7 (13)	N3—C43—H69	120.4
N2—C11—H11	118.6	C44—C43—H69	120.4
C12—C11—H11	118.6	C43—C44—C49	122.0 (13)
C20—C12—C13	122.0 (17)	C43—C44—C45	118.6 (13)
C20—C12—C11	118.0 (15)	C49—C44—C45	119.2 (13)
C13—C12—C11	119.8 (17)	C46—C45—C44	120.1 (12)
C14—C13—C12	119.4 (19)	C46—C45—H67	120.0
C14—C13—H13	120.3	C44—C45—H67	120.0
C12—C13—H13	120.3	C45—C46—C47	117.1 (13)
C13—C14—C15	117 (2)	C45—C46—H66	121.4
C13—C14—H14	121.4	C47—C46—H66	121.4
C15—C14—H14	121.4	C48—C47—C46	126.9 (13)
C16—C15—C14	123 (2)	C48—C47—H65	116.6
C16—C15—H15	118.3	C46—C47—H65	116.6
C14—C15—H15	118.3	C47—C48—C49	114.9 (11)
C15—C16—C20	121 (2)	C47—C48—H64	122.6
C15—C16—H16	119.5	C49—C48—H64	122.6
C20—C16—H16	119.5	C44—C49—C50	115.5 (12)
C18—C17—C20	123.1 (14)	C44—C49—C48	121.7 (13)
C18—C17—H17	118.5	C50—C49—C48	122.8 (13)
C20—C17—H17	118.5	C51—C50—C49	123.6 (13)
N2—C18—C17	117.4 (12)	C51—C50—H62	118.2
N2—C18—C19	116.6 (12)	C49—C50—H62	118.2
C17—C18—C19	125.9 (14)	C50—C51—N3	118.9 (12)
C18—C19—H19A	109.5	C50—C51—C52	126.1 (12)
C18—C19—H19B	109.5	N3—C51—C52	115.0 (12)
H19A—C19—H19B	109.5	C51—C52—H61A	109.5
C18—C19—H19C	109.5	C51—C52—H61B	109.5
H19A—C19—H19C	109.5	H61A—C52—H61B	109.5
H19B—C19—H19C	109.5	C51—C52—H61C	109.5
C12—C20—C17	118.1 (16)	H61A—C52—H61C	109.5
C12—C20—C16	117 (2)	H61B—C52—H61C	109.5
C17—C20—C16	125 (2)	C9—N1—C1	120.5 (10)
C26—C21—C22	120.0 (11)	C9—N1—Ag1	124.8 (8)
C26—C21—S1	118.5 (8)	C1—N1—Ag1	114.5 (8)
C22—C21—S1	121.3 (10)	C11—N2—C18	120.4 (11)
N6—C22—C23	122.4 (11)	C11—N2—Ag1	117.9 (9)
N6—C22—C21	121.6 (12)	C18—N2—Ag1	121.0 (8)
C23—C22—C21	115.4 (12)	C43—N3—C51	120.6 (10)
C24—C23—C22	124.3 (11)	C43—N3—Ag2	114.1 (7)
C24—C23—H95	117.9	C51—N3—Ag2	124.8 (9)
C22—C23—H95	117.9	C33—N4—C41	120.6 (11)
C23—C24—C25	119.1 (11)	C33—N4—Ag2	115.5 (8)
C23—C24—H94	120.4	C41—N4—Ag2	123.5 (9)
C25—C24—H94	120.4	C27—N5—H5NA	124 (5)
C24—C25—C26	119.3 (11)	C27—N5—H5NB	127 (3)
C24—C25—Cl2	121.0 (10)	H5NA—N5—H5NB	109 (4)
C26—C25—Cl2	119.8 (9)	C22—N6—H6NA	105.4
C21—C26—C25	121.8 (11)	C22—N6—H6NB	106.5
C21—C26—H92	119.1	H6NA—N6—H6NB	102.0
C25—C26—H92	119.1	S3—O6—Ag1	159.1 (7)
N5—C27—C28	119.9 (11)	S1—O7—Ag2	172.3 (5)
N5—C27—C32	122.6 (11)	O7—S1—O9	112.8 (5)
C28—C27—C32	117.4 (11)	O7—S1—O8	113.3 (5)
C29—C28—C27	123.0 (11)	O9—S1—O8	112.4 (5)
C29—C28—H99	118.5	O7—S1—C21	104.9 (5)
C27—C28—H99	118.5	O9—S1—C21	105.2 (5)
C28—C29—C30	118.4 (11)	O8—S1—C21	107.5 (5)
C28—C29—H100	120.8	O4—S3—O5	111.9 (6)
C30—C29—H100	120.8	O4—S3—O6	111.9 (7)
C31—C30—C29	120.3 (11)	O5—S3—O6	114.7 (7)
C31—C30—Cl1	119.5 (9)	O4—S3—C32	105.8 (5)
C29—C30—Cl1	120.1 (9)	O5—S3—C32	106.0 (5)
C32—C31—C30	120.7 (10)	O6—S3—C32	105.7 (5)

### Hydrogen-bond geometry (Å, °)

**Table:**

*D*—H···*A*	*D*—H	H···*A*	*D*···*A*	*D*—H···*A*
N6—H6NA···O8	0.83	2.35	2.844 (12)	118
N5—H5NA···O9	0.84 (3)	2.20 (5)	3.006 (13)	159 (11)
N5—H5NB···O5	0.83 (3)	2.48 (6)	2.973 (14)	119 (4)
N6—H6NB···O4^i^	0.84	2.30	3.126 (13)	168

Symmetry codes: (i) *x*, *y*−1, *z*.
